# The involvement of anaesthetists and other healthcare professionals with surgeons in shared decision-making for adult patients contemplating surgery: a systematic review and narrative analysis

**DOI:** 10.1016/j.bjao.2026.100543

**Published:** 2026-03-06

**Authors:** Nisha Sriram, Andreana Glendinning, Timothy J. Stephens, Gemma Hughes

**Affiliations:** 1 The Royal London Hospital, Barts Health NHS Trust, London, UK; 2 Centre of Healthcare Innovation, Policy and Management, University of Leicester, School of Business, Leicester, UK; 3 Critical Care and Perioperative Medicine Research Group, William Harvey Research Institute, Barts and the London School of Medicine and Dentistry, Queen Mary University of London, London, UK

**Keywords:** clinical communication, interprofessional collaboration, multidisciplinary, patient-centred care, perioperative, shared decision-making, surgery, systematic review

## Abstract

**Background:**

Recognising the medical and surgical complexity of certain patient populations, perioperative pathways are evolving to incorporate multidisciplinary team perspectives, alongside surgeons, in shared decision-making. We aimed to synthesise the evidence base for multidisciplinary team shared decision-making.

**Methods:**

We performed a systematic literature review accessing bibliographic databases (MEDLINE (using OVID), EMBASE, PsycINFO, Web of Science, CINAHL, and ProQuest), the international prospective register for systematic reviews (PROSPERO), the International Standard Randomised Control Trial Number Registry, and the King’s Fund Library Database.

**Results:**

Of 8727 citations, 271 were eligible for full-text review and 29 included in the final narrative synthesis. A wide variety of non-surgical healthcare professionals (e.g. anaesthetist, geriatrician, radiation oncologist, intensivist, specialist nurse, physiotherapist, psychologist, and social worker) and others (including peer support) participated in perioperative shared decision-making. Consultations were mainly structured sequentially or concomitantly with surgical consultations. Patients and clinicians generally responded positively to multidisciplinary team shared decision-making. Where measured, decisional quality was reported to have improved, and decisional conflict reduced. Studies of high-risk patients indicated that multidisciplinary team shared decision-making was more likely to result in non-operative management. We identified five features of these modes of multidisciplinary team shared decision-making consultation: identification of relevant experts required, expert multidisciplinary assessment, thorough consideration of alternative treatments, coordinated patient support, and additional consultation time.

**Conclusions:**

Some surgical populations, as a function of the complexity of their medical, surgical, or both condition(s), would benefit from multidisciplinary team shared decision-making. Further research is required to understand the optimal organisation of this approach and the impact on patient and systems outcomes.

**Systematic review protocol:**

PROSPERO (CRD42025636328).

Shared decision-making between patients and healthcare professionals is widely recognised as important in promoting patient-centred perioperative care.^1^ Precise definitions of shared decision-making vary within the literature. However, unifying elements include collaborative participation between at least two parties (i.e. patient and healthcare professional), to share information, weigh up treatment options, and agree on actions.^2^, ^3^, ^4^ Using shared decision-making in elective surgery can decrease surgical intervention rates, reduce decisional conflict, and improve decision quality.^5^, ^6^, ^7^

Reflecting wider demographic trends, the volume of surgery performed on an ageing, increasingly high-risk population is steadily growing.^8^ Older patients are more likely to live with frailty and multi-morbidity.^9^ While shared decision-making is available to all patients, it is particularly important for older, high-risk cohorts where the risk of post-surgical disability and death is significant.^10^ Surgery may result in lack of therapeutic benefit or an unacceptable functional capacity for patients. A shared decision-making conversation with exploration of the patient’s values can build mutual understanding between patients and healthcare professionals regarding surgical goals.^11^

Patients often commence their perioperative journey by meeting with a surgeon. However, the extent to which surgeons can facilitate shared decision-making may be constrained by the goals of the consultation and the values of the participants. An interview study of surgeons’ views towards high-risk surgical decision-making found that surgeons prioritised assessing risk and setting expectations over involving patients as active participants in decision-making.^12^ In practice, a study of perioperative patient–surgeon consultations observed that surgical communications leant towards obtaining informed consent over facilitating collaborative decision-making.^13^ If decisions about surgery are approached by surgeons and patients with a ‘fix-it’ mental model, this can undermine deliberation about *whether* surgery (particularly high-risk surgery) should happen.^14^ Finally, surgeons, as experts in surgical treatments, may be unable to counsel patients comprehensively on the potential alternatives to surgery. When it comes to comparing risks and benefits of different treatments, surgeons, like any physician, might be biased towards the treatments they offer.^15^

Shared decision-making may be constrained by the sequencing of consultations within traditional perioperative pathways. Patients are initially seen by surgeons, who then refer them to preoperative anaesthetist-led assessment clinics if surgery is agreed upon. Anaesthetic assessments may identify novel medical comorbidities, risks, and uncertainties with perioperative implications. It is questionable if holistic shared decision-making (between anaesthetist and patient) is possible when an established decision to choose surgery exists. In principle, the decision to have surgery should be reversible throughout the preoperative process. However, the anaesthetist’s contribution is contextualised within the pre-existing decision to operate and may be influenced by hierarchical relationships with surgeons.^16^^,^^17^ In practice, ‘clinical momentum’ means that surgical interventions are difficult to pause by patients and healthcare professionals once a surgical problem is identified.^18^

In recognition of the limitations of traditional perioperative structures, pathways are being re-engineered to improve patient experience, outcomes, and efficiency.^19^ This involves increasing the variety of specialist clinician input and encouraging greater multidisciplinary contributions to shared decision-making.

A broad spectrum of non-surgical healthcare professionals contribute perioperatively. Anaesthetists as perioperative physicians both lead and participate within multidisciplinary teams to deliver perioperative patient care.^20^, ^21^, ^22^, ^23^, ^24^ Another alternative model of care for older people is geriatrician-led, incorporating comprehensive geriatric assessment (CGA) methodology.^25^^,^^26^ CGA is a structured multidisciplinary assessment of a range of health domains to create a holistic care plan.^27^ Other healthcare professionals with an impact on perioperative shared decision-making within the literature include primary care physicians^28^ and medical and radiation oncologists.^29^ More widely, decision coaching, undertaken by a range of professionals including counsellors, nurses, and psychologists, has been found to be effective in preparing patients for involvement in shared decision-making for a range of health decisions, including but not limited to surgery.^30^

Clearly, perioperative shared decision-making is a collaborative endeavour requiring contribution from a wide range of healthcare professionals including physicians, nurses, and allied health professionals alongside surgeons. Whilst there is a substantial body of literature on models and components of shared decision-making (e.g. see Bomhof-Roordink and colleagues^31^), there has not been a review, to date, mapping the identities and impact of non-surgical healthcare professionals in shared decision-making for surgery.

The primary aim of this review was to ascertain which healthcare professionals, in addition to surgeons, contribute to shared decision-making about surgery, and to identify the different modes of involvement for those healthcare professionals. Overall, the review aimed to understand how anaesthetists, perioperative physicians, and other healthcare professionals were involved in shared decision-making with patients about surgery and the feasibility of their involvement. Secondary aims were to identify how different healthcare professionals interacted with patients in relation to shared decision-making in the perioperative period and to assess ways in which the participation of different healthcare professionals affected patients’ experiences and outcomes of shared decision-making about surgery. This study does not focus exclusively on the older patient, as shared decision-making is best practice for all ages, however, when studies explicitly refer to the older patient, this is reflected within the results.

## Methods

This study was a systematic review and narrative analysis of published data up to September 2025. The review protocol was registered on the international prospective register of systematic reviews (PROSPERO) in January 2025 (registration number CRD42025636328). Searches were conducted in January 2025, with title/abstract screening completed in May 2025. Searches were re-run in October 2025. We followed the Preferred Reporting Items for Systematic reviews and Meta-Analyses (PRISMA) guidelines^32^ (see Supplementary Table 1).

### Research questions

We asked:

1. Who was involved in perioperative shared decision-making in addition to surgeons?
2. How was the involvement of professionals organised?
3. What impact did this additional involvement have? (e.g. on patient outcomes, patient experience, clinician satisfaction, and system outcomes).

### Search strategy

We designed a comprehensive search strategy with the guidance of a specialist librarian. We searched bibliographic databases: MEDLINE (using OVID), EMBASE, PsycINFO, Web of Science, CINAHL, and ProQuest. We searched the international prospective register for systematic reviews (PROSPERO), the International Standard Randomised Control Trial Number Registry, and the King’s Fund Library Database for grey literature. We also consulted with the Expert Advisory Group for the study, a group comprised of eight healthcare professionals and lay members, to nominate papers. We conducted forward and backward citation tracking from included papers. Our search consisted of the terms ‘decision making’, ‘surgery’, ‘patients’, and ‘clinicians’, using the Boolean operator ‘and’ (see Supplementary File 1, Supplementary Tables 2–10).

### Inclusion and exclusion criteria

All studies of adults (aged 18 yr and over) considering surgical treatment were included. We defined surgery as an invasive procedure requiring an incision, performed by a member of a recognised surgical speciality.^33^ We defined healthcare professionals as persons accredited with a recognised professional body, with permission to conduct and supervise relevant healthcare activities.^34^

We excluded studies about decisions for surgery featuring children or adolescents under the age of 18 yr. Studies were excluded if they focused exclusively on the surgeon–patient decision-making process, or it was impossible to ascertain the identity of the other healthcare professionals involved. Studies without patient representation were excluded (e.g. studies of multidisciplinary team decision-making without patients present). Studies of surrogate or proxy decision-making were excluded. Studies about attitudes to or perceptions of shared decision-making and studies about developing or testing decision aids were excluded. We limited the search to studies published in English. We excluded editorials, case reports, and protocols.

### Study selection and data extraction

**Fig 1:**
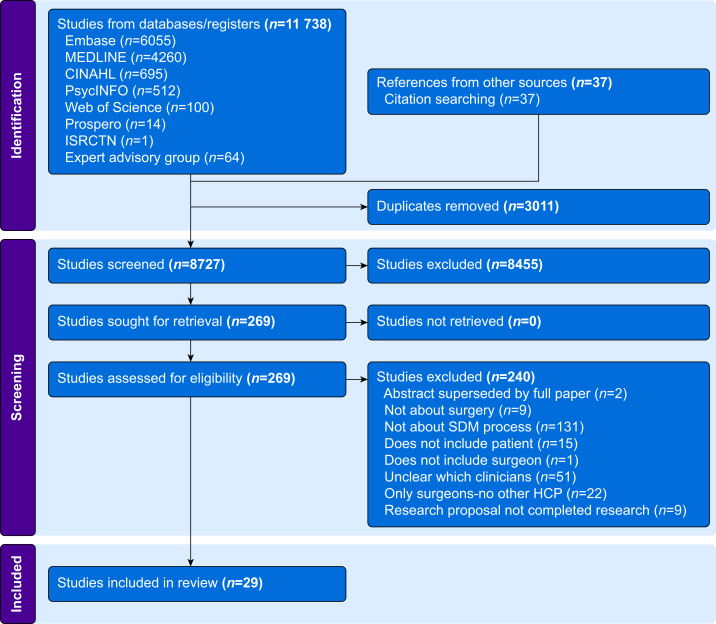
Study selection procedure. HCP, healthcare professional; SDM, shared decision-making.

**Fig 2:**
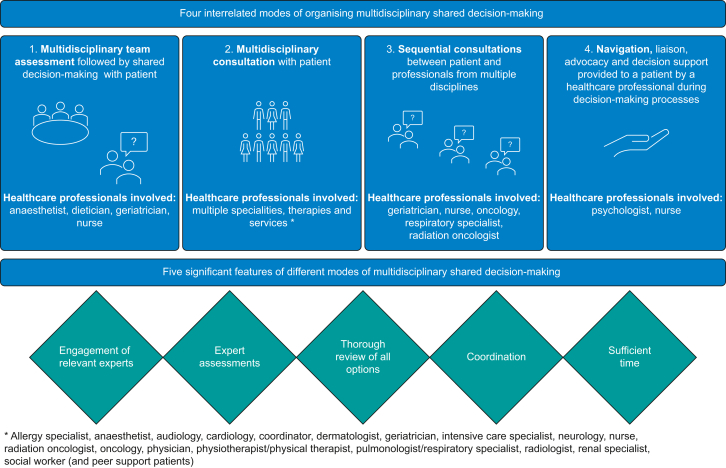
Visual summary of modes and features of multidisciplinary shared decision-making.

Articles were screened in two phases, title and abstract followed by full-text review. Each article was screened independently by two reviewers from the team of four authors (AG, GH, NS, TJS). Disagreements were resolved by discussion with at least one other author. Data extraction was conducted independently by NS and GH and results were compared; disagreements were resolved by consensus discussion. Data extracted were: study design, clinical setting, patient and clinician characteristics, nature of shared decision-making process, and outcomes. Covidence systematic review software was used to organise the screening of abstracts and full texts, resolution of conflict, and creation of the data extraction template.^35^ Automative features used were the de-duplication function and population of the PRISMA diagram (see Fig. 1 for diagram of study selection). All screening, extraction, and template design processes were manually completed by the reviewers (Fig. 2).

### Quality appraisal

Two reviewers (NS and GH) independently critically appraised the methodological quality and risk of bias of included studies using the Mixed Methods Appraisal Tool (MMAT)^36^ (see Supplementary File 1, Supplementary Table 11). Using the number of no/yes/cannot tell responses to the questions within the MMAT as a guide, the authors categorised the quality of papers as low (two or fewer ‘yes’ responses) medium (three or more ‘yes’ responses but with at least one ‘no’ or ‘cannot tell’), or high (‘yes’ to all). For the quantitative papers, low and medium quality would be considered a high or ‘of concern’ risk of bias, respectively. Disagreements were resolved by discussion with one other author (TJS). Most articles were of low (*n*=11) or medium quality (*n*=12) with six rated as high quality. No studies were excluded based on the quality appraisal.

### Narrative synthesis

Because of the heterogeneity of methods, measures, and variable quality of the included studies, a narrative synthesis approach was used across the full set of papers.^37^ We had intended to complete sub-group analyses of high-risk and low-risk procedures and high-risk patients undergoing emergency surgery. However, insufficient data precluded the undertaking of these sub-group analyses. Analysis involved creating a descriptive summary table to compare study populations, clinical service settings, surgical interventions, healthcare professional identities, and modes of organising shared decision-making pathways. First, we compared similar populations/service settings. Then we identified common modes of organising shared decision-making across the full set of papers. Finally, we compared measured outcomes.

## Results

### Identification of studies

The initial searches yielded a total of 11 738 articles, with 8727 remaining once duplicates were removed. Some 8455 studies were excluded for ineligibility at title and abstract screening. Of the 271 included for full-text review, 240 articles were excluded. Most of these were not about the process of shared decision-making (e.g. patients were not involved) or were not sufficiently clear on which healthcare professionals were involved in addition to the surgeon. After full-text review, 29 articles remained, two papers reported on different aspects of the same study meaning there were 28 different services/interventions included. We excluded two abstracts (conference proceedings or posters) on interventions that were reported in full in included articles but included seven abstracts about interventions where there was no other published evidence. These abstracts provided limited data available to inform this review.

### Study characteristics

The studies were predominantly conducted in high-income countries: UK (13 papers reporting on 12 interventions), Europe (one from France, one from Germany, and one from the Netherlands), North America (four from the USA and three from Canada), New Zealand (two), Australia (two), and the Republic of Korea (one). One study was conducted in an upper middle-income country (China, one). The earliest study was published in 2004, with one other study published within that decade. Most studies (*n*=18) were published since 2020, with nine studies published between 2010 and 2019. There were two randomised control trials (RCT), one of which was a pilot and six qualitative studies. The remainder were primarily observational and cross-sectional studies of interventions, including retrospective cohort studies and quality improvement studies.

### Patient cohorts and service settings

Fewer than half of the studies (*n*=13) focused on services for older patients, variously described as frail, high-risk, multi-morbid, or all three. Four of these were focused on colorectal surgery,^38^, ^39^, ^40^, ^41^ one on cardiac surgery,^42^ one on urologic surgery,^43^ and one on surgery after hip fracture.^44^ The remaining six covered general/unspecified surgery, with one of those focusing on emergency general surgery,^45^ two studies including both elective and emergency surgery,^26^^,^^46^ and the remaining three covering elective surgery.^47^, ^48^, ^49^

There were four studies of decisions about treatment options including surgery for prostate cancer patients,^50^, ^51^, ^52^, ^53^ four papers about postmastectomy breast reconstruction for breast cancer patients,^54^, ^55^, ^56^, ^57^ two articles about an intervention for neurosurgery decisions for trigeminal neuralgia patients,^58^^,^^59^ and one study for decisions about each of the following: patients with confirmed or suspected non-melanoma skin cancer,^60^ patients with non-arthritic hip pain,^61^ patients with vestibular schwannoma,^62^ patients with abdominal aortic aneurism,^63^ patients with early-stage non-small-cell lung cancer,^64^ and patients with inflammatory bowel disease (IBD)^65^ (see Table 1 for a summary of studies).

**Table 1:** Summary of studies. CBAG, coronary artery bypass graft; CGA, comprehensive geriatric assessment; MDT, multidisciplinary team; POPS, perioperative care for older people having surgery; SDM, shared decision-making.

Author (yr)	Study design	Patient population	Treatment decisions	Professionals involved in addition to surgeons	Models of multidisciplinary shared decision-making	Location
Ali and colleagues (2021)^47^	Observational	High-risk surgical patients, older	Surgical procedures (not specified, described as complex and highly invasive)	Anaesthetist	Collaborative anaesthetist-led, shared decision-making model of care providing multidisciplinary preoperative assessment and optimisation and postoperative follow up	Australia
Asfour and colleagues (2015)^60^	Observational	Patients with confirmed or suspected non-melanoma skin cancer	Surgery, radiotherapy, photodynamic/topical/clinical observation	Oncologist (radiotherapist), dermatologists	Multidisciplinary clinic (with patient)	UK
Bowler and colleagues (2016)^43^	Cohort study	Older urological surgery patients	Surgery	Geriatrician, anaesthetist	Multidisciplinary perioperative assessment	UK
Braude and colleagues (2017)^57^	Qualitative	Patients with breast cancer diagnosis in one breast, or known genetic carriers or strong family history of breast cancer	Risk reducing mastectomy, bilateral mastectomy/contralateral prophylactic mastectomy	Psychologist	Referral to psychologist within MDT	Australia
Brown-Taylor and colleagues (2022)^61^	Randomised controlled trial	Patients with non-arthritic hip pain	Physical therapy or hip arthroscopy	Physical therapist	Independent evaluation of patient by surgeon and physical therapist. Surgeon-PT discussion. Three-way SDM (surgeon, PT, patient)	USA
Causarano and colleagues (2015)^54^	Randomised controlled trial	Postmastectomy breast cancer patients	Breast reconstruction	Breast reconstruction clinical nurse specialist, patient peer support and social worker	Pre-consultation education group—same day as surgical consultation	Canada
Colombo and colleagues (2023)^62^	Retrospective cohort study	Patients with vestibular schwannoma	Stereotactic radiosurgery (SRS)/radiotherapy or microsurgery	Neurooncologist (radiotherapy), specialist nurse, audiology	Interdisciplinary counselling: semi-adversarial counselling process with nurse supporting patient	UK
Davies and colleagues (2024)^63^	Prospective cohort study	Patients with abdominal aortic aneurysm (AAA)	Vascular surgery	Consultant vascular anaesthetist, consultant vascular radiologist (if required), specialist nurses, dedicated coordinator	Multi-Professional Clinic for AAA. Patients referred from screening. Patients meet with all health professionals together	UK
Dhesi and Swart (2016)^46^	Observational	Older people with long-term medical comorbidities	Elective surgery and emergency surgery (POPS)	Anaesthetist, geriatrician, generalist and specialist nurses, physiotherapists, occupational therapists, and dieticians	Specialist preoperative consultations: (1) surgical risk assessment; (2) SDM clinic and proactive care	UK
Gainer and colleagues (2024)^42^	Prospective cohort study	Patients ≥65 yr referred for cardiac surgery (non-urgent)	CBAG or valve surgery	Decisional coach (cardiac surgery patient navigator—experienced cardiac surgery nurse)	Formalised SDM pathway, risk calculator, decision aid, and decisional coach—who liaised with surgeon	Canada
Golden and colleagues (2017)^64^	Qualitative	Patients with early-stage non-small-cell lung cancer	Surgical resection or stereotactic body radiotherapy (SBRT)	Radiation oncologist, pulmonologists, thoracic oncology nurse	Individual SDM consultations	USA
Harcourt and colleagues (2016)^55^	Qualitative	Women considering breast reconstruction postmastectomy	Breast reconstruction	Psychologist (PEGASUS coach)	PEGASUS—goal setting with a psychologist, which is taken into consultation with surgeon	UK
Jackson and colleagues (2024)^38^	Retrospective cohort study	Older people with colorectal cancer	Elective colorectal surgery	Anaesthetist, geriatrician/specialist physician	SDM pathway: holistic assessment in a single outpatient clinic collectively with surgeon, anaesthetist and specialist physician, followed by values-based discussion	New Zealand
Lal and colleagues (2023)^26^	Quality improvement and prospective observational (mixed methods)	High-risk older perioperative patients	Elective and emergency surgery	Geriatrician	POPS: multidisciplinary geriatrician-led CGA and optimisation service	UK
Leonard and colleagues (2025)^48^	Qualitative	Older patients	Surgery	Primary care geriatricians	Framework for support from primary care geriatricians	USA
Li and colleagues (2025)^65^	Observational	Inflammatory bowel disease (IBD) patients	Range of IBD treatments including surgery	Specialist nurse (multidisciplinary centre also included internal medicine, pathology, radiology, nutrition, laboratory medicine, pharmacy, and dermatology)	Multidisciplinary IBD diagnosis and treatment centre with specialist nurse appointed. Nurse was patient advocate, provided psychological support, assessed patients’ values and preferences, discussed uncertainties, coordinated multidisciplinary meetings, and acted as decision coach	China
Madsen and colleagues (2009)^50^	Observational	Newly diagnosed prostate cancer patients	Multiple treatment options including radical prostatectomy	Multidisciplinary clinic including radiation oncologist (and medical oncologist for high-risk patients) plus advanced practice nurse	Multidisciplinary clinic with nurse role to support and educate patients, and coordinate care during the decision-making process without apparent treatment bias	USA
Norridge and colleagues (2024)^45^	Cross-sectional study	Frail patients, facing complex treatment decisions	Emergency general surgery	Older persons clinical nurse specialist	Surgical liaison service, SDM led by clinical nurse specialist	UK
Omundsen and colleagues (2020)^49^	Quality improvement	High-risk, older patients	Elective surgery	Anaesthetist, intensive care specialist plus other specialists as needed (cardiology, respiratory, renal, neurology, and allergy)	Complex decision pathway, referred by surgeons, triaged by anaesthetists (investigations and specialist input requested), then clinic with anaesthetist and intensivist (2-h appointment—1 h with patient/family)	New Zealand
Patrikidou and colleagues (2018)^51^	Cross-sectional study	Newly diagnosed, non-metastatic prostate cancer patients	Surgery, non-surgical treatment, watchful waiting	Radiation oncologist	Prostate Cancer Multidisciplinary Clinic: interdisciplinary consultations, successive consultations (45 min) with radiation oncologist and urological surgeon then tumour board discussion on same day which establishes final treatment plan	France
Poole and colleagues (2022)^58^	Cross-sectional study	Trigeminal neuralgia patients	Medication, neurosurgery	Physician	Joint consultation clinic	UK
Schostak and colleagues (2004)^52^	Cross-sectional study	Patients with prostate cancer	Radical surgery or radiotherapy	Radiation oncologists	Interdisciplinary consulting service	Germany
Singhota and colleagues (2022) ^59^	Cross-sectional study	Trigeminal neuralgia patients	Medical or surgical treatment	Physician	Joint clinic	UK
Staiger and colleagues (2023)^39^	Retrospective cohort study	Older people undergoing major colorectal surgery: patients with comorbidities requiring optimisation	SDM re risks *vs* benefits of surgery, optimisation	Geriatrician, anaesthetist	Multidisciplinary perioperative clinic for older persons. Referred by surgeon or anaesthetist after notes review, joint assessment by consultant anaesthetist and geriatrician, involvement of surgical team if surgical options reviewed	UK
Than and colleagues (2023)^40^	Cross-sectional study	Older, frail patients undergoing major colorectal surgery	Elective colorectal surgery	Geriatrician, anaesthetist	Combined geriatric and anaesthetist pre-assessment clinic	UK
Thera and colleagues (2018)^53^	Qualitative	Localised prostate cancer patients	External beam radiotherapy, surgery (radical prostatectomy), brachytherapy, active surveillance	Nurse navigator	Nurse navigator consultation to discuss options post diagnosis, followed by treatment or further specialist consultation	Canada
Tollow and colleagues (2021)^56^	Qualitative	Women considering breast reconstruction postmastectomy	Breast reconstruction	Psychologist (PEGASUS coach)	Trained coach plus tool to be used in surgical consultation	UK
van der Zwaard and colleagues (2020)^44^	Retrospective cohort study	Older (70+) patients with hip fracture	Surgery, non-surgical management	Geriatrician	Preoperative CGA with SDM	The Netherlands
Yang and colleagues (2022)^41^	Cross-sectional study	Frail patients with colorectal cancer	Surgery, adjuvant therapy, palliative therapy	Medical oncologist, radiation oncologist, radiologist, geriatrician, coordinating nurse	Geriatric multidisciplinary oncology clinic. CGA conducted before clinic, then panel meet with patient and family	Korea

### Models of multidisciplinary shared decision-making and professionals involved

The studies predominantly discussed multidisciplinary, interdisciplinary, or joint approaches to shared decision-making where patients had perioperative consultations with multiple non-surgical healthcare professionals at different timepoints. The majority of these presented new models being tested or introduced, for example as preoperative quality improvement projects. We found that some studies referred to formal models of shared decision-making, whereas others described pathways or approaches. We describe *models* of care where those were specified, and categorise our findings according to broader *modes* of shared decision-making which were apparent across formalised models of care and more generic pathways.

Models for older people included anaesthetist-led, geriatrician-led, and joint anaesthetist/geriatrician approaches. Anaesthetist-led approaches were: an Australian shared decision-making model that included perioperative multidisciplinary assessments (the Westmead Hospital Perioperative Medicine Service),^47^ a complex decision pathway (CDP) that involved anaesthetist, intensivist, and other specialist input in New Zealand,^49^ and a surgical risk assessment and shared decision-making clinic in the UK (at Torbay Hospital).^46^ Geriatrician-led models of care included: a UK multidisciplinary geriatrician-led CGA and optimisation service (the Perioperative Care for Older People Service or POPS at Guys’ and St Thomas’ Hospital) ,^26^^,^^46^ CGA and shared decision-making with patients with hip fracture in a trauma centre in the Netherlands,^44^ a geriatric multidisciplinary oncology clinic (GMOC) at Seoul National University Bundang Hospital, Korea,^41^ and a framework to assist geriatricians in primary care (in the US context) to support older adults navigate perioperative care including supporting decision-making.^48^ Anaesthetists and geriatricians were jointly involved in multi/interdisciplinary perioperative assessments and clinics at the University Hospital of North Tees in the UK,^43^ at Waikato Hospital in New Zealand,^38^ in the Colchester Older Persons’ Evaluation for Surgery (COPES) clinic in the UK,^39^ and at Southend Hospital, UK.^40^

Two studies of services for older people focused on the role of specialist nurses; a formalised shared decision-making pathway for patients 65 yr and older referred to the Department of Cardiac Surgery in the Nova Scotia Health Authority in Canada^42^ and a clinical nurse specialist who led shared decision-making for emergency general surgery in Southampton Hospital in the UK.^45^

For prostate cancer patients, multidisciplinary clinics/interdisciplinary consulting services were provided in the USA,^50^ France,^51^ and Germany^52^ which facilitated discussions between patients, surgeons, and radiation oncologists about the alternative treatments available. This gave patients the opportunity to discuss non-surgical treatment options (e.g. radiotherapy) with the specialist physicians who would provide those treatments, just as they would discuss surgery with surgeons. A Canadian service introduced a decision-making programme lead by a nurse navigator who would discuss treatment options with prostate cancer patients and facilitate consultations with specialists as needed.^53^ The nurse role was also central in the US study in supporting patients and coordinating care throughout decision-making.^50^

There were four articles about three interventions designed to support patients with decisions about breast reconstruction postmastectomy. The interventions were: a pre-consultation education group intervention in Canada which involved five patients meeting with a surgeon, a nurse specialist, a social worker, and two peer support patients^54^; a UK intervention known as PEGASUS (Patient Expectations and Goals: Assisting Shared Understanding of Surgery), comprising a psychologist acting as a decisional coach in a structured consultation before meeting with their surgeon^55^^,^^56^; and an Australian study of the role of a psychologist in supporting decision-making.^57^

Two papers reported on a multidisciplinary trigeminal neuralgia clinic in London, UK which involved patients meeting with neurosurgeons and physicians together.^58^^,^^59^

Other ways of organising shared decision-making were: oncologists and dermatologists providing multidisciplinary clinics with plastic surgeons for patients with skin cancer^60^; physical therapists consulting with orthopaedic surgeons for patients with non-arthritic hip pain^61^; neuro-oncologist, specialist nurse, and audiologist consulting with neurosurgeon (skull base) for patients with vestibular schwannoma in Manchester, UK^62^; consultant vascular anaesthetist, consultant vascular radiologist, specialist nurses, and a dedicated coordinator providing a multidisciplinary clinic for patients with abdominal aortic aneurism in Sheffield, UK^63^; radiation oncologist, pulmonologist, thoracic oncology nurse, and thoracic surgeons participating in shared decision-making with patients with lung cancer in the USA^64^; and a specialist nurse coordinating multidisciplinary care for patients with IBD in Zhejiang, China which included professionals from internal medicine, surgery, pathology, radiology, nutrition, laboratory medicine, pharmacy, and dermatology.^65^

A total of 26 different roles, speciality areas, or both were identified across the studies that contributed to multidisciplinary shared decision-making (see Table 2): allergy specialist, anaesthetist (including consultant vascular anaesthetist), audiology, cardiology, coach (provided by nurse and psychologist), coordinator, dermatologist, dietician/nutrition, geriatrician, intensive care specialist/intensivist, laboratory medicine, neurology, nurse (including advance practice nurse, clinical nurse specialist and generalist and in roles that included advocate, navigator, and coordinator), occupational therapist, oncologist (including radiotherapist, neurooncologist, urologic oncologist, and medical oncologist), pathologist, patient peer support, pharmacy, physician (including internal medicine and specialist physician), physiotherapist/physical therapist, psychologist, pulmonologist/respiratory specialist, radiation oncologist, radiologist, renal specialist, and social worker.

**Table 2:** summary of roles/specialist input included.

Role/specialism	Study
Allergy specialist	^49^
Anaesthetist	38, 39, 40^,^^42^^,^^46^^,^^47^^,^^49^^,^^63^
Audiology	^66^
Cardiology	^49^
Coach fulfilled by nurse, psychologist	^42^^,^^55^^,^^56^^,^^65^
Coordinator dedicated role	^63^
Dermatologist	^60^^,^^65^
Dietician/nutrition	^46^^,^^65^
Geriatrician	^26^^,^^38^, ^39^, ^40^, ^41^^,^^43^^,^^44^^,^^46^^,^^48^
Intensive care specialist	^49^
Laboratory medicine	^65^
Neurology	^49^
Nurse including specialist, generalist and role as navigator, coach, and advocate	^26^^,^^41^^,^^42^^,^^45^^,^^50^^,^^53^^,^^54^^,^^62^, ^63^, ^64^, ^65^
Occupational therapist	^46^
Oncology including radiotherapy, neuro-oncology, urologic, and medical	^41^^,^^50^^,^^60^^,^^62^
Pathology	^65^
Patient peer support	^54^
Pharmacy	^65^
Physician specialist, internal medicine	^38^^,^^58^^,^^59^^,^^65^
Physiotherapist/physical therapist	^46^^,^^61^
Psychologist	55, 56, 57
Pulmonologist/respiratory specialist	^49^^,^^64^
Radiation oncologist	^41^^,^^51^^,^^52^^,^^64^
Radiologist	^41^^,^^63^^,^^65^
Renal specialist	^49^
Social worker	^54^

### Modes of organising multidisciplinary shared decision-making

We identified four different modes of organising multidisciplinary interactions between patients and healthcare professionals:

1. Multidisciplinary assessment and shared decision-making processes: different healthcare professionals were involved throughout the perioperative pathway (named healthcare professional roles/specialisms were: anaesthetist, dietician, and geriatrician, nurse).^26^^,^^40^^,^^43^^,^^44^^,^^46^^,^^47^
2. Multidisciplinary group consultations: sometimes referred to as clinics or panels, patients met with several healthcare professionals simultaneously (named healthcare professional roles/specialisms were: allergy specialist, anaesthetist, audiology, cardiology, coordinator, dermatologist, geriatrician, intensive care specialist, neurology, nurse, radiation oncologist, oncology, physician, physiotherapist/physical therapist, pulmonologist/respiratory specialist, radiologist, renal specialist, and social worker (and in one intervention also with peer support patients)).^38^^,^^39^^,^^41^^,^^49^^,^^52^^,^^54^^,^^58^, ^59^, ^60^, ^61^, ^62^, ^63^
3. Multidisciplinary sequential consultations where patients met with healthcare professionals in turn (named healthcare professional roles/specialisms were: geriatrician, nurse, oncology, pulmonologist/respiratory specialist, and radiation oncologist).^48^^,^^50^^,^^51^^,^^64^
4. Navigation, liaison, advocacy or decision support, involving one or more meetings between patients and a healthcare professional (psychologist or nurse) who acted as a navigator or coach.^42^^,^^45^^,^^50^^,^^53^^,^^55^^,^^56^^,^^57^^,^^64^^,^^65^

These arrangements were not mutually exclusive. For example, some care pathways combined individual and group consultations, such as the independent evaluation of patients with non-arthritic hip pain by a physical therapist and then a surgeon, followed by a three-way meeting between physical therapist, surgeon, and patient.^61^ In addition, within navigational support pathways, the navigator accompanied patients to consultations, for example a specialist nurse advocated for and supported patients with vestibular schwannoma in an interdisciplinary counselling process with surgeon and oncologist.^62^

We identified five features of these modes of consultation and support that were reported as being significant in delivering multidisciplinary shared decision-making: identification of relevant experts required, expert multidisciplinary assessment, thorough consideration of alternative treatments, coordinated patient support, and extended consultation time. The goals of shared decision-making interventions varied based on the necessities of the studied groups (e.g. clinical assessment for older patients, access to multidisciplinary specialists for discussion when varied treatment options existed). We provide a diagrammatic summary of the modes and features of multidisciplinary shared decision-making identified in this review in Figure 2.

Multidisciplinary assessment was a critical element of pathways for older high-risk patients. A complex decision pathway (CDP) is an example of a structure where surgeons identified and referred patients requiring specialist assistance with decision-making input. Patients were then triaged by an anaesthetist. When clinical investigations and other specialist input had been collated, each patient was booked for a 2-h clinic appointment with an anaesthetist and an intensivist. The clinicians spent 1 h reviewing the patient, undertaking risk scoring and consulting with other specialists and the remainder with the patient (and their support person/carer) in shared decision-making discussions.^49^ An alternative pathway structure with a different approach was the geriatrician-led service in the elective and emergency setting.^26^^,^^44^^,^^46^ Via the employment of CGA framework, patients were evaluated and targets for intervention were identified. This informed the shared decision-making conversations led by geriatricians with patient (and their support person/carer). For high-risk patients, these assessments considered whether surgery was appropriate in relation to their medical comorbidities and frailty, functional capabilities, social circumstances, and cognitive impairments. In addition, thorough perioperative assessment could identify targets for intervention and optimisation. Multidisciplinary assessment was also incorporated into pathways for other patients requiring input from allied health professionals (e.g. a physical therapist and surgeon evaluating patients with non-arthritic hip pain).^61^

Shared decision-making for patient cohorts where multiple treatment options existed, required access and discussion with the expert clinicians who provided the alternative treatment options to surgery (e.g. radiotherapy). One approach was the ‘semi-adversarial’ counselling process demonstrated in a shared decision-making clinic for patients with vestibular schwannoma. Patients were counselled by a surgeon advocating for surgery and an oncologist advocating for radiosurgery or radiotherapy. Patients, having accessed two opinions, were supported by specialist nurses to select their treatment.^62^ An alternative approach designed for patients with prostate cancer involved sequential consultations over 45 min with a radiation oncologist and urological surgeon, followed by a multidisciplinary tumour board meeting not attended by the patient. Patients could obtain specialist opinions both directly and indirectly to make treatment decisions.^51^ Clinicians recognised the challenges of communicating accurate information about risks and benefits of treatment they did not deliver.^64^ Multidisciplinary clinics were seen to enhance active patient involvement and confidence in their care plans.^51^

Logistical coordination was necessary to organise multidisciplinary activities, as was support and advocacy for patients during the process. A dedicated coordinator was in place for a multi-professional clinic for abdominal aortic aneurism care,^63^ in other services specialist nurses provided ongoing coordination and support for patients^41^^,^^42^^,^^44^; nurses also had important roles in surgical liaison^45^ and risk assessment.^46^

The time taken to organise and provide multidisciplinary shared decision-making was noted in studies from different settings, with lengthy clinics and consultations necessary for the complex assessment and decision-making process involved.^49^^,^^51^^,^^52^^,^^60^

### Outcomes of multidisciplinary shared decision-making

The main outcomes assessed by studies were: changes to pathways; the feasibility, acceptability and use of different approaches from clinicians’ perspectives; patients’ experiences of decision-making processes; decisional conflict and quality; treatment choices; the impact on services; patient reported outcomes; morbidity; and mortality.

Generally, pathway changes and new models introduced were reported to be feasible and positively received by clinicians,^26^^,^^46^^,^^50^^,^^54^^,^^55^^,^^57^^,^^58^^,^^65^ with the need to have buy-in and consistency from the whole team noted in relation to a new approach to decision-making for breast reconstruction.^56^ Two studies were of existing, rather than new, models of care. A qualitative study of clinicians’ experiences of lung cancer care found some limitations in their ability to practice shared decision-making, with a greater focus given to information giving as opposed to eliciting patients’ preferences and values^64^ and a study of how primary care geriatricians supported their patients in navigating perioperative care in the USA found this work has been underreported in the literature, and uncompensated.^48^

Patients’ experiences of multidisciplinary shared decision-making were generally reported as being positive,^26^^,^^51^, ^52^, ^53^^,^^55^^,^^56^^,^^60^ empowering,^43^ and leading to improved understanding.^42^^,^^58^ Decisional quality was reported to have improved, as measured by the SDM-Q-9^42^^,^^45^; decisional conflict (as measured by the Ottawa scale) was reduced^42^^,^^54^^,^^61^ and decision self-efficacy was improved.^54^

Treatment choices were assessed in 14 studies. Some studies of high-risk patients indicated that multidisciplinary shared decision-making was more likely to result in choices of non-operative management and reported this as a positive way of avoiding non-beneficial surgery and reducing risk of adverse outcomes.^38^^,^^40^^,^^43^^,^^44^^,^^47^^,^^49^ A pilot RCT to evaluate the feasibility and effect of a group intervention for decisions about postmastectomy breast reconstruction also found fewer patients chose to consent to breast reconstruction after the intervention.^54^

Other studies reported little effect on treatment choices. Decisions to undergo percutaneous coronary intervention or medical management did not significantly differ from decisions to undergo cardiac surgery,^42^ and whilst patients showed increased interest in physical therapy for non-arthritic hip pain, this was not statistically significant.^61^

Some studies indicated that patients do not necessarily chose the least invasive options after multidisciplinary shared decision-making.^59^^,^^62^ Modification of treatment plans and better treatment selection were indicated in some studies^51^^,^^52^ as was the production of individualised care plans.^46^

There was no clear evidence about patient outcomes related to service use, with both increases^43^ and decreases^39^ in length of stay reported, similarly both shorter^55^ and longer^60^ waiting times were reported. There was no conclusive evidence about readmission rates.

There were no statistically significant effects reported of multidisciplinary shared decision-making on quality of life (measured through EQ-5D-3L) or anxiety and depression (using the Hospital Anxiety and Depression scale) ^42^ and no evidence of effects on mortality or morbidity.^38^^,^^47^

## Discussion

We categorised the non-surgical healthcare professionals reported in the included studies into 26 different roles working within the perioperative pathway, with anaesthetists, geriatricians, and nurses predominating (see Table 2). Four main modes of organising multidisciplinary shared decision-making were identified, with widespread heterogeneity between studies. Participation of non-surgical healthcare professionals in perioperative shared decision-making was deemed as feasible and received positively by clinicians and patients. However, we found no consistent evidence of impact of multidisciplinary shared decision-making on other relevant measures namely treatment choices, quality of life, and patient service use outcomes. Further, there was no evidence about the optimum mode or modes of organising multidisciplinary shared decision-making.

There have been no previous studies, to our knowledge, of the roles of different healthcare professionals in multidisciplinary shared decision-making for surgery. By extracting data from studies of interventions and modes of shared decision-making about surgery for adults, we have identified a range of people who contribute, their individual roles, and the different models that they work within. Multidisciplinary shared decision-making is well-established within a variety of fields including renal dialysis,^66^ community palliative care,^67^ and oncology.^68^ This review provides new knowledge about how multidisciplinary shared decision-making can be extended into the surgical context. It adds to previous work that has established the importance of multidisciplinary evaluation for perioperative patient evaluation, optimisation, and shared decision-making.^69^

A strength of this review is the disparateness of surgical populations involved. However, this limits our ability to conduct a meta-analysis. The findings are further constrained by the relatively weak evidence and the low or medium quality of many of the studies. Our searches for literature were limited by being unable to access non-English language research. Whereas the cultural and religious values of both healthcare professionals and patients can influence their engagement with shared decision-making,^70^ studies were predominantly conducted in high-income countries and without a particular focus on cultural or religious diversity. We therefore cannot comment on this important aspect of shared decision-making.

We described shared decision-making as a collaboration between patient and healthcare professionals. Barriers for participation in shared decision-making are known to exist at the individual (patients, healthcare professionals) and systemic level. Previous research has identified patient-related barriers, including imbalances in knowledge and power with healthcare professionals, and the inability to consider alternative non-surgical options.^71^^,^^72^ Healthcare professionals have previously recognised that surgical culture and inadequate integration of working between professionals impacted on shared decision-making.^73^ Furthermore, hierarchical complexities and professional boundaries have been described between surgeons, physicians (anaesthetists), and nurses, which may be relevant.^74^, ^75^, ^76^ Systems barriers included time pressures and overwhelmed professional capacity to deliver shared decision making.^71^ Multidisciplinary decision-making adapted to specific clinical contexts could potentially overcome some of these barriers. Through the incorporation of peer supporters, psychologists, and navigators, patients may have access to approachable professionals for assistance and clarification of their goals. Patients who consult with non-surgical specialists (e.g. oncologist) can find greater confidence in choosing non-surgical alternatives. Multidisciplinary working can offer clinicians greater opportunities to work across professional boundaries, and build interpersonal relationships. Systemic barriers need to be addressed by organisational investment, for example ring-fenced clinical encounters. Identifying precisely which healthcare professionals should engage in multidisciplinary shared decision-making is not possible because of differences in requirements for various clinical scenarios. For example, patients choosing between surgery and chemotherapy may require engagement from surgeons and oncologists. Frail patients considering surgery need a geriatrician-led perioperative evaluation. However, this is an area requiring further contextual research.

Other areas requiring study include elucidating the specific roles of nurses in shared decision-making, and comparison of modes of organising multidisciplinary shared decision-making in terms of patient- (e.g. acceptability) and systems-centred outcomes (e.g. cost).

## Authors’ contributions

Conceptualisation: NS, TJS, GH

Formal analysis, investigation: all authors

Data curation: NS

Writing—original draft preparation: NS, GH

Writing—reviewing and editing: AG, TJS

Funding acquisition, supervision: TJS, GH

Approved the final version to be published and agree to be accountable for all aspects of the work: all authors

## Funding

The National Institute for Health and Care Research (NIHR) Programme Grants for Applied Research (PGfAR) Programme Development Grants (NIHR207604).

## Declarations of interest

The authors declare that they have no conflicts of interest.
